# The Global Atlas of Helminth Infection: Mapping the Way Forward in Neglected Tropical Disease Control

**DOI:** 10.1371/journal.pntd.0000779

**Published:** 2010-07-27

**Authors:** Simon Brooker, Peter J. Hotez, Donald A. P. Bundy

**Affiliations:** 1 London School of Hygiene and Tropical Medicine, London, United Kingdom; 2 Kenya Medical Research Institute-Wellcome Trust Research Programme, Nairobi, Kenya; 3 George Washington University, Washington, District of Columbia, United States of America; 4 Sabin Vaccine Institute, Washington, District of Columbia, United States of America; 5 The World Bank, Washington, District of Columbia, United States of America; Yale School of Public Health, United States of America

The recent commitment of the Obama administration to establish the Global Health Initiative, which is expected to increase to over US$100 million annually for neglected tropical disease (NTD) control, provides the most significant investment and opportunity for the global control of NTDs to date [1], [2]. These investments, together with commitments by the British Department for International Development, the World Bank, and several key private philanthropies, including the Bill & Melinda Gates Foundation, must be guided by a strong evidence-based approach. First, the problem, and the resources required to tackle it, need to be clearly quantified. Second, mass drug administration (MDA) should be optimally targeted to communities with the highest prevalence of infection and presumed greatest morbidity [3], [4]. Furthermore, for diseases targeted for elimination, including lymphatic filariasis (LF) and onchocerciasis, it will become increasingly important to determine whether MDA can be stopped, and, if so, when and where. In the case of schistosomiasis, as control is scaled up, there is the additional requirement of determining when and where to shift praziquantel treatment from once per year to less frequent intervals.

## New Diagnostic Tools

Better diagnostic tools and new methods of surveillance provide more affordable and realistic opportunities to improve the planning, monitoring, and evaluation of NTD control. Diagnosis of onchocerciasis was previously based on detection of microfilariae in skin snips, but this invasive technique is gradually being replaced by antibody-based tests, which can provide a simple and rapid method of diagnosis [5], [6]. The development of a simple and rapid antigen detection test for *Wuchereria bancrofti* antigenaemia, based on the immuno-chromatographic test (ICT card test), revolutionized LF surveys since it avoided the need to collect blood at night and the time-consuming preparation and examination of blood slides [7]. Ongoing efforts are investigating similar rapid antigen detection tests for schistosomiasis [8]. This new generation of diagnostics provides a sound foundation for developing reliable, up-to-date maps of the distribution of different NTDs to guide and target resources efficiently. Without such maps, the journey of NTD control will be difficult.

## Mapping of NTDs

In the past, national reporting on NTDs has been incomplete and unreliable because of weak disease surveillance systems, often necessitating dedicated surveys to be undertaken. Perhaps the first attempt to develop evidence-based maps of any NTD was that of the African Programme for Onchocerciasis Control (APOC), which developed the rapid epidemiological mapping of onchocerciasis (REMO) approach to quickly and cheaply identify priority areas for community-directed treatment with ivermectin (CDTI) and estimate the number of individuals requiring treatment [9]. This approach stratifies a country into areas that are suitable and unsuitable for transmission, based on known geographic factors, and within suitable areas implements rapid assessment of communities by screening individuals for onchocercal nodules. In Africa, a total of 23 countries have been mapped for onchocerciasis. As countries have been successful in controlling onchocerciasis, many are now conducting new REMO assessments to provide updated maps. For LF, the rapid geographical assessment of Bancroftian filariasis (RAGFIL) method [10] using ICT card tests enabled large-scale assessments of the boundaries of filaria-endemic areas for identifying areas requiring MDA (with albendazole and ivermectin or diethylcarbamazine-citrate) [11]. In 2009, all endemic countries in the World Health Organization (WHO)-defined regions of the Eastern Mediterranean, South-East Asia, and Western Pacific have completed mapping, whilst in Africa, 37 of 39 endemic countries have completed or are in the process of mapping, with only Chad and Eritrea yet to start [12]. In the case of schistosomiasis, one of the first steps of the Schistosomiasis Control Initiative (SCI) in designing country programmes was to conduct national prevalence surveys of schistosomiasis to identify communities and districts requiring mass treatment with praziquantel [13], [14]. Outside SCI-supported countries, national surveys of schistosomiasis and soil-transmitted helminth (STH) infections are often scarce and ad-hoc, with the exception of some dedicated surveys, such as those supported by the Carter Center [15], [16] and most recently by countries supported by the NTD Initiative of the US Agency for International Development (USAID) [17]. Despite these efforts, there remains a considerable mapping requirement to support global NTD control. Also, it is little appreciated that as control is successful in reducing transmission, there will be an increasing requirement to conduct mapping surveys to assess whether to stop MDA or switch to less frequent treatment.

## New Mapping Technologies

Future data collection efforts should take advantage of recent advances in recording and processing data and converting data into maps. Increasingly, surveys utilize electronic data capture systems, mainly based on the use of personal digital assistants (PDAs) but also small laptops, to enter data at the point of collection [18] and almost instantaneously transmit the information to a central database using mobile phone technology [19]. Once data have been collected and collated, geographical information systems (GIS) provide a ready framework in which to manage and display the data. The distribution of NTDs is particularly sensitive to climatic and environmental factors because of the vulnerability of vectors, intermediate hosts, and free-living stages. The GIS frameworks allow ready comparison between disease patterns and environmental data, while remote sensing (RS) technologies can use high-resolution satellite data to provide estimates of such variables as temperature, vegetation (as a proxy for various environmental factors), and humidity [20]. The relationships between observed infection patterns and environmental factors can be investigated using both traditional and spatially explicit statistical approaches [21], allowing the spatial distributions of infection prevalence to be predicted in unsurveyed areas. Such analyses are increasingly adopting a Bayesian approach to statistical inference that provides a robust method for measuring uncertainty in prediction [22], [23]. Finally, it is perhaps worth emphasizing that this journal is itself an example of the power of the new technologies, since the open-access format permits the data, analyses, and predictions to be published in a way that maximizes access to the information.

## Current Global Mapping Resources

Although many surveys of different NTDs have been conducted and many more are planned, the survey data are only useful if available in a form that is accessible to policy-makers and the managers of public health programmes. At present the most detailed maps available are for onchocerciasis, LF, and schistosomiasis. For onchocerciasis, the country-level REMO maps, which highlight the areas where CDTI is needed, are made available through APOC's Web site [24]. For schistosomiasis, the most complete global resource remains the 1987 *Atlas of the Global Distribution of Schistosomiasis* [25], now available online [26]. For each country, a map is presented that shows the presence or absence of schistosomiasis. Although these maps represent an important early source of information, the data are at best 23 years old and do not include the wealth of prevalence data collected more recently.

The preventive chemotherapy (PCT) databank was developed by WHO as a tool to map progress on the implementation of NTD control efforts targeting LF, schistosomiasis, and STH infections [27]. The PCT databank includes country profiles that present the estimated population at risk of infection and requiring PCT and, where available, data on treatment coverage for each NTD [28]. However, the PCT databank only includes data as reported to WHO and excludes survey information from other sources, such as data collected by academic researchers, nongovernmental organizations (NGOs), and other partners. The data are presented at administrative level 1 (province or region) and the derived maps inevitably cannot capture the fine-scale heterogeneity of infection patterns, so they tend to overestimate the numbers of individuals requiring treatment.

## A Global Atlas of Helminth Infection

In an attempt to provide open access to up-to-date information on schistosomiasis and STH infections, a project has been undertaken to develop a Global Atlas of Helminth Infection (GAHI) [29], [30]. The overall goal of this project is to provide an open-access, global information resource on the distribution of STH and schistosomiasis, with the specific aims of 1) describing the global distribution and prevalence of infection of each species and 2) highlighting geographical areas for which further survey information is required. The maps are grounded in structured searches of the formal and grey literature for suitable survey data that are then collated into a single database, according to specified inclusion criteria. The eligible surveys are geo-positioned using electronic sources and maps are then developed using GIS. To date, more than 10,000 prevalence surveys have been identified, catalogued, and mapped. The potential usefulness of the data to identify “at risk” populations for which data are scarce is enhanced by using Bayesian model-based geostatistics [23] to predict the prevalence of infection with each species in as yet unsurveyed areas.

The GAHI Web site will be launched on August 12, 2010 at http://www.thiswormyworld.org/. The Web site allows users to visualize the assembled data and models. Three types of maps are presented for every country where these infections occur: (i) a S*urvey Data Map* showing the prevalence of infection based on survey data; (ii) a *Predictive Risk Map* showing the probability that infection prevalence warrants MDA, according to recommended WHO thresholds; and (iii) a C*ontrol Planning Map* showing which districts require MDA treatment or where further surveys would be helpful in defining risk.

As the URL suggests, inspiration for this project comes from the work of the American parasitologist Norman Stoll, who, during his 1946 presidential address to the American Society of Parasitologists, posed the question, just how much human helminthiasis is there in the world? The resultant paper, *This Wormy World* [31], was the first systematic attempt to measure the worldwide impact of human parasitism by helminths and provides the foundation for subsequent attempts to define the burden of NTDs [32]. Building on the GAHI, an ongoing project is developing a Global Atlas of Trachoma, expanding earlier attempts to the map the global distribution of trachoma [33]. This work is intended to provide an evidence base for allocating resources for trachoma control, including surgery and administration of Zithromax [35].

## Conclusion

Accurate and up-to-date maps of different NTDs can help improve the precision of decision-making in NTD control. They can help increase the reliability of estimates of disease burden, for example, as part of the ongoing revision of the Global Burden of Disease study [34]. The maps can also establish a baseline against which to measure the impact of NTD control efforts. Finally, they can provide an important planning tool for national control programmes. Considerable effort is required to develop an integrated NTD atlas, necessitating cooperation and collaboration of the different NTD communities. We hope the Global Atlas of Helminth Infection will encourage this interaction and help progress towards an open access Global Atlas of NTDs.

## References

[1] United States Government (2010). Implementation of the Global Health Initiative: consultation document.. http://www.pepfar.gov/ghi/index.htm.

[2] White House (2009). Statement by the President on Global Health Initiative.. http://www.whitehouse.gov/the_press_office/Statement-by-the-President-on-Global-Health-Initiative.

[3] Brooker S, Utzinger J (2007). Integrated disease mapping in a polyparasitic world.. Geospatial Health.

[4] Baker MC, Mathieu E, Fleming FM, Deming M, King JD (2010). Mapping, monitoring, and surveillance of neglected tropical diseases: towards a policy framework.. Lancet.

[5] Lipner EM, Dembele N, Souleymane S, Alley WS, Prevots DR (2006). Field applicability of a rapid-format anti-*Ov*-16 antibody test for the assessment of onchocerciasis control measures in regions of endemicity.. J Infect Dis.

[6] Burbelo PD, Leahy HP, Iadarola MJ, Nutman TB (2009). A four-antigen mixture for rapid assessment of *Onchocerca volvulus* infection.. PLoS Negl Trop Dis.

[7] Molyneux DH (2009). Filaria control and elimination: diagnostic, monitoring and surveillance needs.. Trans R Soc Trop Med Hyg.

[8] Stothard JR (2009). Improving control of African schistosomiasis: towards effective use of rapid diagnostic tests within an appropriate disease surveillance model.. Trans R Soc Trop Med Hyg.

[9] Noma M, Nwoke BEB, Nutall I, Tambala PA, Enyong P (2002). Rapid epidemiology mapping of onchocerciasis (REMO): its application by the African Programme for Onchocerciasis Control (APOC).. Ann Trop Med Parasitol.

[10] World Health Organization (2005). Monitoring and epidemiological assessment of the programme to eliminate lymphatic filariasis at implementation unit level.

[11] Gyapong JO, Kyelem D, Kleinschmidt I, Agbo K, Ahouandogbo F (2002). The use of spatial analysis in mapping the distribution of bancroftian filariasis in four West African countries.. Ann Trop Med Parasitol.

[12] World Health Organization (16 October 2009). Global programme to eliminate lymphatic filariasis.. Wkly Epidemiol Rec.

[13] Clements ACA, Lwambo NJS, Blair L, Nyandindi U, Kaatano G (2006). Bayesian spatial analysis and disease mapping: tools to enhance planning and implementation of a schistosomiasis control programme in Tanzania.. Trop Med Int Health.

[14] Clements AC, Garba A, Sacko M, Touré S, Dembelé R (2008). Mapping the probability of schistosomiasis and associated uncertainty, West Africa.. Emerg Infect Dis.

[15] Hopkins DR, Eigege A, Miri ES, Gontor I, Ogah G (2002). Lymphatic filariasis elimination and schistosomiasis control in combination with onchocerciasis control in Nigeria.. Am J Trop Med Hyg.

[16] King JD, Eigege A, Richards F, Jip N, Umaru J (2009). Integrating NTD mapping protocols: Can surveys for trachoma and urinary schistosomiasis be done simultaneously?. Am J Trop Med Hyg.

[17] Sturrock HJW, Picon D, Sabasio A, Oguttu D, Robinson E (2009). Integrated mapping of neglected tropical diseases: epidemiological findings and implications for integrated control for Northern Bahr-el-Ghazal State, Southern Sudan.. PLoS Negl Trop Dis.

[18] Shirima K, Mukasa O, Schellenberg JA, Manzi F, John D (2007). The use of personal digital assistants for data entry at the point of collection in a large household survey in southern Tanzania.. Emerg Themes Epidemiol.

[19] Aanensen DM, Huntley DM, Feil EJ, al-Own F, Spratt BG (2009). EpiCollect: linking smartphones to web applications for epidemiology, ecology and community data collection.. PLoS ONE.

[20] Hay SI, Tatem AJ, Graham AJ, Goetz SJ, Rogers DJ (2006). Global environmental data for mapping infectious disease distribution.. Adv Parasitol.

[21] Diggle PJ, Ribeiro PJ (2007). Model-based geostatistics.

[22] Basáñez MG, Marshall C, Carabin H, Gyorkos T, Joseph L (2004). Bayesian statistics for parasitologists.. Trends Parasitol.

[23] Magalhães RJS, Clements ACA, Patil AP, Gething PW, Brooker S (2010). The applications of model-based geostatistics in helminth epidemiology and control.. Adv Parasitol.

[24] World Health Organization (2010). African Programme of Onchocerciasis Control. Country profiles.. http://www.who.int/apoc/countries/en.

[25] Doumenge JP, Mott KE, Cheung C, Villenave D, Chapuis O (1987). Atlas of the global distribution of schistosomiasis.

[26] World Health Organization (2010). Atlas of the global distribution of schistosomiasis.. http://www.who.int/wormcontrol/documents/maps/en.

[27] World Health Organization (2010). Preventive chemotherapy databank.. http://www.who.int/neglected_diseases/preventive_chemotherapy/databank/en/index.html.

[28] World Health Organization (2010). Preventive chemotherapy databank. Country profiles for countries in the WHO African Region.. http://www.who.int/neglected_diseases/preventive_chemotherapy/databank/databank_CP_AFRO/en/index.html.

[29] Brooker S, Rowlands M, Haller L, Savioli L, Bundy DAP (2000). Towards an atlas of human helminth infection in sub-Saharan Africa: the use of geographical information systems (GIS).. Parasitol Today.

[30] Brooker S, Kabatereine NB, Smith JL, Mupfasoni D, Mwanje MT (2009). An updated atlas of human helminth infections: the example of East Africa.. Int J Health Geographics.

[31] Stoll NR (1947). This wormy world.. J Parasitol.

[32] de Silva NR, Brooker S, Hotez PJ, Montresor A, Engels D, Savioli L (2003). Soil-transmitted helminth infections: updating the global picture.. Trends Parasitol.

[33] Polack S, Brooker S, Kuper H, Mariotti S, Mabey D, Foster A (2005). Mapping the global distribution of trachoma.. Bull World Health Organ.

[34] International Trachoma Initiative (2010). Zithromax® in the elimination of blinding trachoma. a program manager's guide.

[35] Institute for Health Metrics and Evaluation (2010). Global burden of disease study.. http://www.globalburden.org/.

